# Cognitive Enrichment in Practice: A Survey of Factors Affecting Its Implementation in Zoos Globally

**DOI:** 10.3390/ani11061721

**Published:** 2021-06-09

**Authors:** Belinda A. Hall, David M. McGill, Sally L. Sherwen, Rebecca E. Doyle

**Affiliations:** 1 Animal Welfare Science Centre, Faculty of Veterinary and Agricultural Sciences, The University of Melbourne, Parkville 3052, Australia; ssherwen@zoo.org.au (S.L.S.); rebecca.doyle@unimelb.edu.au (R.E.D.); 2 Mackinnon Group, Faculty of Veterinary and Agricultural Sciences, The University of Melbourne, Werribee 3030, Australia; dmcgill@unimelb.edu.au; 3 Wildlife Conservation and Science, Zoos Victoria, Parkville 3052, Australia

**Keywords:** animal enrichment, barriers to adoption, captive vertebrate management, cognition, practice change

## Abstract

**Simple Summary:**

Cognitive enrichment gives animals the opportunity to challenge themselves and control aspects of their environment through problem solving. Despite the known benefits of giving captive animals cognitive enrichment, not much is known about how it is used in zoos. This survey found that staff within zoos think that cognitive enrichment is very important for the welfare of animals. However, its use is not widespread. While some animal groups like carnivores commonly receive cognitive enrichment, animals like fish and reptiles are overlooked. Time and financial support were found to be common factors that had a high impact on the use of cognitive enrichment, while zookeeper interest was said to be important for its success. Findings suggest that animal keepers, who are most often involved in enrichment programs, need to be better supported to deliver cognitive enrichment. Enrichment programs need to be prioritized with the creation of job roles specifically for enrichment or increased time and training given to keepers to carry out these duties.

**Abstract:**

Information on the practical use of cognitive enrichment in zoos is scarce. This survey aimed to identify where cognitive enrichment is being used while identifying factors that may limit its implementation and success. Distributed in eight languages to increase global range, responses to this survey (*n* = 177) show that while agreement on what constitutes cognitive enrichment is poor, it is universally perceived as very important for animal welfare. Carnivores were the animal group most reported to receive cognitive enrichment (76.3%), while amphibians and fish the least (16.9%). All animal groups had a percentage of participants indicating animal groups in their facility were not receiving cognitive enrichment when they believe that they should (29.4–44.6%). On average, factors relating to time and finance were rated most highly in terms of effect on cognitive enrichment use, and keeper interest was the highest rated for effect on success. Results of this study indicate that cognitive enrichment is perceived as important. However, placing the responsibility of its development and implementation on animal keepers who are already time-poor may be impeding its use. A commitment to incorporating cognitive enrichment into routine husbandry, including financial support and investment into staff is needed from zoos to ensure continued improvement to captive animal welfare.

## 1. Introduction

Over the last few decades, our understanding of what comprises good animal welfare has evolved. An example of this is the expansion of the long-accepted Five Freedoms model to the Five Domains model [1]. This updated model highlights the importance of affective states for welfare and quality of life [1]. The Five domains model is widely accepted and used to underpin policy in governing bodies like the Zoos and Aquarium Association Australasia [2]. To support positive affective states animals must be given the opportunities to express natural behaviors [3]. There is evidence of a broad range of cognitive capabilities in animals including, but not limited to, problem solving [4], conceptualization [5] and tool use [6], as well as the drive to be challenged, as demonstrated by contrafreeloading [7]. More recent research demonstrating the cognitive ability of non-mammal species has continued to alter the way we view and value animal intelligence [8,9]. Inadequate opportunities to experience challenge have been linked to underdeveloped competence, negative emotional states, reduced behavioral expression and decreased healing [10]. As such, a lack of cognitive challenge in captive environments is, at best a missed opportunity to increase welfare, and at its worst, a source of negative welfare [11].

Environmental enrichment can facilitate basic levels of cognitive challenge in barren environments or add additional levels of complexity into already simulating ones. Broadly, environmental enrichment is defined as something which causes “an improvement in the biological functioning of captive animals resulting from modifications of their environment” [12]. This environmental modification is often achieved through the implementation of programs involving one or more elements from the following known enrichment categories: nutritional, sensory, structural, social (human or animal) and/or occupational (psychological or physical challenge) [13]. Environmental enrichments have been shown to have physiological benefits, such as decreased mortality and morbidity [14] and increased activity [15,16], as well as psychological benefits, seen through the reduction of stereotypies [17], the prevention of depression and anxiety [18] and reduced behaviors associated with boredom [19]. In the early 2000s, environmental enrichment was reported to be predominately delivered to captive mammals, as opposed to other taxa, with cognitive enrichment being the least common enrichment given [20]. This bias towards mammals, and in particular primates, has been maintained in cognitive research resulting in a reduced ability to create appropriate cognitive enrichment for other species [21].

For enrichment to be classed as cognitive, it is suggested that the enrichment must fulfil the following criteria “(1) engages evolved cognitive skills by providing opportunities to solve problems and control some aspect of the environment, and (2) is correlated to one or more validated measures of wellbeing” [22]. Research supports the welfare benefits of presenting cognitive enrichment to a range of species through positive alteration of behavior such as increased curiosity and contact with novel objects in goats [23], decreased feather plucking in parrots [24], increased time spent voluntarily engaging with enrichment under water in dolphins [25] and reduced inactivity in chimpanzees [26]. Evidence of problem solving and generalization abilities have also been shown in lizards [27] and fish [28]. While this list is not at all exhaustive, it demonstrates the diversity of species, which can benefit from cognitive enrichment.

A survey looking into the use of traditional forms of enrichment in zoos found that a lack of time was the main factor restricting its implementation [29]. This 2010 survey by Hoy et al. [29] did not investigate cognitive enrichment, however, as successful implementation of cognitive enrichment requires additional time to identify and supply the appropriate type and level of challenge [21], a lack of time may impede cognitive enrichment use as well. The perception that cognitive enrichment is too complex to deliver has been raised as another possible barrier to its use [30]. The cause of this perception has not been investigated but may stem from previous exposure to highly technical cognitive enrichment such as those developed for primates involving touch screens [31,32] or a lack of understanding of what can be constituted as cognitive enrichment. This means that even though reviews of the literature detail positive welfare benefits from cognitive enrichment [21,30,33], barriers to successful implementation may be present within zoos. If there is a disparity between the literature and what is delivered in practice, the cause of this needs to be investigated so that animal welfare standards can be maintained to the best of our ability and cognitive challenge can be routinely incorporated into husbandry practices.

This study aimed to identify how cognitive enrichment is valued and routinely incorporated in zoos globally, as well as identify factors that may influence its use. We hypothesized the factors affecting the use and success of cognitive enrichment would vary between regions and job role due to differences in perception of cognitive enrichment and resource availability.

## 2. Materials and Methods

### 2.1. Ethics Approval

This study was approved by the University of Melbourne’s Faculty of Veterinary and Agricultural Sciences Human Ethics Committee, ethical review number 1953643.3.

### 2.2. Survey

This survey was developed based on gaps in the literature regarding the practical use of cognitive enrichment in zoos as well as barriers to enrichment implementation highlighted by Hoy et al., 2010. The survey was comprised of eight general topics: 1. attitudes towards enrichment and welfare; 2. current use of different types of enrichment; 3. factors affecting the use of different types enrichment; 4. logistics of implementing enrichment; 5. classification of cognitive enrichment; 6. personal beliefs and attitudes 7. job satisfaction; and 8. general demographics. After the survey was compiled it was piloted with seven zookeepers, and modified based on their feedback, before being widely circulated. With the aim to understand the way zoos use cognitive enrichment globally, the survey was translated into eight languages (English, French, German, Spanish, Japanese, simplified Chinese, Korean and Italian). Translation was done by native speakers, that were also fluent in English, most of whom had a background in animal welfare.

The final survey that was distributed consisted of 47 questions. The majority of were answered using either multiple-choice responses or Likert scales (1–7) with the most negative response at 1 and the most positive at 7.

Before starting the survey respondents were given a definition of enrichment “Environmental enrichment may be defined as; a behavioural husbandry principle that aims to promote psychological and physiological well-being in captive animals” [34]. A question requiring respondents to class items into the categories ‘Cognitive enrichment’, ‘Not cognitive enrichment’ or ‘Not enrichment’ was asked before a definition of cognitive enrichment was given to identify what respondents considered as cognitive enrichment. Respondents were then asked to answer questions relating to how important they thought different types of enrichment were, how often they were used, who was involved in enrichment delivery and asked to rate the significance of factors that limit their use of both traditional enrichment and cognitive enrichment. A definition of cognitive enrichment was then given in the survey which was as follows “Cognitive enrichment has been defined as enrichment which allows animals to solve problems and/or control their environment while having a positive effect on their welfare”, which is an abbreviated version of the definition from Clark in 2011 [22]. Questions regarding which animal groups received/should receive cognitive enrichment as well as how a range of factors impacted the success of cognitive enrichment were then asked.

### 2.3. Respondents

The targeted demographic were staff from captive animal facilities who worked either directly with animals, or within the facility but in a non-contact role with the animals. Examples of these positions include animal keepers, management in animal-related roles, management in non-animal-related roles and grounds keeping staff.

Respondents were recruited through a variety of means: personal contacts, emails sent directly to zoos, advertisement on Twitter and Facebook groups with an interest in the areas of enrichment, zoos, husbandry, and welfare. Due to the availability of translators only zoos located in English and German-speaking countries were emailed directly. A list of zoos worldwide was found using an internet search and 190 located in Germany and 339 zoos from English speaking countries were invited to participate in the survey.

A focused campaign was also used to recruit staff at one focal organization in Australia, and these responses were classified as their own cohort. This cohort was recruited though their staff email addresses, and the survey was distributed by the organization themselves.

### 2.4. Data Handling and Statistics

#### 2.4.1. Data Cleaning

A total of 177 respondents were included in the analysis. From the 368 times the link was open, 188 respondents completed the majority of questions (51% completion rate). Ten respondents were removed due to missing region information and a single participant was removed due to being the only respondent from their region.

When classifying sex, *n* = 6 was grouped as NA, combining “Other” (*n* = 1) with those who did not answer the question (*n* = 5).

Questions that focused on factors influencing the use of enrichment were worded differently for respondents that worked directly or indirectly with animals. These were amalgamated into 10 factors for analytical purposes (Table 1).

#### 2.4.2. Analysis

All analysis was performed using the statistical software R (sourced from R Foundation for Statistical Computing, Vienna, Austria) [35]. Descriptive statistics were used to report on means, medians, proportions, and standard errors that were calculated using CRAN (Comprehensive R Archive Network) package ‘expss’. Linear regression analysis was used to look for differences in response between different groups factoring in sex, region, and role type. Traditionally linear regression models are not used for Likert scale data, as was done here, due to the data not being normally distributed. However the validity of these assumptions have been investigated and it has been found that parametric tests are robust enough to handle the ordinal data from Likert scales and give accurate results with sufficient data size, even when violating the assumption of normal distribution [36].

## 3. Results

### 3.1. Demographic Summary

The survey was translated into eight languages (English, French, German, Spanish, Japanese, simplified Chinese, Korean and Italian) so that respondents from a variety of regions were able to participate. A breakdown of which countries participated by region are as follows; Asia: Singapore, South Korea, Japan, Bangladesh; Europe: England, Gibraltar, Germany, The Netherlands, France; North America: United States of America; Oceania: Australia, New Zealand, Papua New Guinea; South and Central America: Argentina, Costa Rica, Colombia, Brazil, Chile; Focal zoo: employed by focal zoo in Australia. Full demographic information can be found in Table 2.

### 3.2. What Is Cognitive Enrichment?

**Figure 1:**
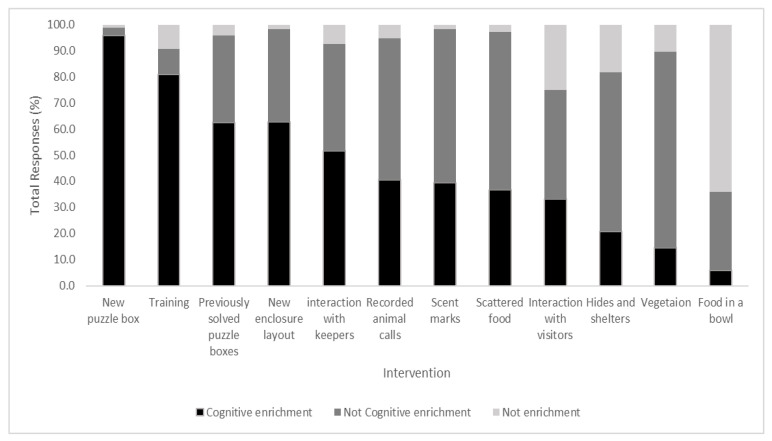
Percentage of respondents who classed each intervention as ‘Cognitive enrichment’, ‘Not Cognitive enrichment’ or ‘Not enrichment’.

Respondents were asked to categorize common enrichments into “Cognitive enrichment” “Not cognitive enrichment” or “Not enrichment” before they were given a definition of cognitive enrichment (Figure 1). There was no consensus on category for any of the interventions, new puzzle boxes had the most agreement with the majority of respondents categorizing them as cognitive enrichment (95.9%). Training was the next most likely enrichment to be categorized as cognitive (80.9%).

### 3.3. Importance of Cognitive Enrichment

When asked how important each enrichment type was for the welfare of captive animals, the median response was that all forms of enrichment were >4 on the likert scale, indicating they were of at least moderate importance. The median score of importance for cognitive enrichment was 7 out of a possible 7 (7 = very important; Table 3).

### 3.4. Creation and Delivery of Enrichment

Overall, 90% of respondents indicated that zookeepers were, in some way, in charge of the creation and delivery of enrichment. The next group most commonly involved were volunteers (43%). Only 24% of respondents reported that dedicated welfare/enrichment workers were involved in the creation and/or delivery of enrichment at their facility. However, there was a regional difference in this, with 67% of South/Central American respondents reporting dedicated welfare/enrichment workers involved in enrichment creation and/or delivery.

### 3.5. Use of Cognitive Enrichment

**Figure 2:**
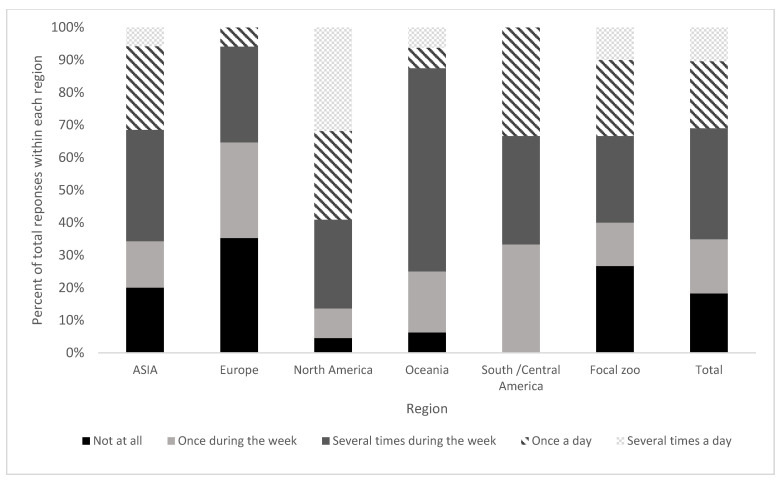
The frequency of cognitive enrichment use within each region, displayed as a percentage of the total responses from each region.

Cognitive enrichment was provided more than once in the week by 65.1% of respondents (sum of responses ‘several times during the week’, ‘once a day’ and ‘several times a day’; Figure 2). By comparison, feeding enrichment was reported as being provided more than once a week by 85.8% of respondents. Similarly, human-animal enrichment was reported as being provided more than once a week by 79.5% of respondents.

Variation in frequency of cognitive enrichment use was seen between regions (Figure 2). Over a third of respondents from the EU/UK reported that cognitive enrichment was not provided in their zoo at all (35.3%). The focal zoo also had many reporting that cognitive enrichment was not given (26.7%). North Americans self-reported the highest frequency of use with 31.8% stating that cognitive enrichment was used more than once a day in their facilities.

Cognitive enrichment use varied between animal groups and regions (Table 4). Carnivores, including big cats, dogs, meerkats, bears, hyena, etc., were reported to receive cognitive enrichment most often (76.3%). In comparison, fish and amphibians were reported to receive cognitive enrichment the least (16.9%). All animal groups had respondents indicating that despite their opinion the animals should be receiving cognitive enrichment, it was not currently provided in their facility. Reptiles received the highest number of respondents advocating for the use of cognitive enrichment where none was currently being used (44.6%).

Questions of which animal groups received cognitive enrichment in the respondents’ facility, and which animal groups they felt should be receiving cognitive enrichment, but did not, were not mutually exclusive. This means that respondents could select both if some species within an animal group receive cognitive enrichment and others were believed to need cognitive enrichment, but currently were not getting it. As a result, totals for both carnivores and other primates surpass 100%.

### 3.6. Factors Affecting the Use of Cognitive Enrichment

Time constraints were perceived to have the most significant effect on the use of cognitive enrichment across all regions and roles. The factors ‘time to observe animal response’, ‘limited funds’, ‘number of animals’ and ‘workload for others’ were all scored as having an impact on the implementation of cognitive enrichment (all > 4/7 on the likert scale; Table 5).

Perceptions around the influence of ‘limited funds’, ‘importance of enrichment in the workplace’, ‘restrictions/regulation on enrichment’, and ‘past enrichment failures’ on the implementation of enrichment differed depending on the role of the respondent (Table 5). Management believed that funding and the importance of enrichment to the workplace did not impact the implementation of cognitive enrichment as much as those working directly or indirectly with animals. The factor of ‘limited funds’ with also rated higher by females than males (means of 5.1 ± standard error 0.17 and 4.5 ± standard error 0.28 respectively). No other results were influenced by non-regional demographics.

There were several regional effects on factors affecting enrichment. South/Central America believed the number of animals significantly affected the provision of cognitive enrichment, compared to Oceana and the focal zoo. The workload for others was rated as a more important factor in Asia than North America and Oceania. The importance of enrichment to management was also much more influential in Asia than other regions. Asia also reported ‘limited knowledge’, ‘limited training’ and ‘concerns around unnatural enrichment’ as being important factors affecting use of cognitive enrichment, rating them well above some other regions.

### 3.7. Factors Affecting the Success of Cognitive Enrichment

All factors, other than visitor interest and animal sex, were scored as having an effect on the success of cognitive enrichment (all > 4/7 on the likert scale; Table 6). The individual factor of ‘keeper interest’ was the most influential factor in the success of cognitive enrichment. Variation between region existed with respondents from Asia differing from at least one other region in five out of eight of the factors.

## 4. Discussion

This survey showed that respondents within zoos believe cognitive enrichment plays an important role in maintaining good animal welfare, but there is weak consensus on what constitutes cognitive enrichment. Furthermore, while deemed to be important across species groups, use of cognitive enrichment is not universally widespread among different species. In line with our hypothesis, there were effects of region and role on the perceptions on cognitive enrichment, although these differences were not as marked as predicted.

The widespread agreement that cognitive enrichment is one of the most important types of enrichment for the welfare of captive animals is not reflected in its reported use. The responses from this study show that cognitive enrichment is not supplied to all species, nor is it the most frequently supplied enrichment type. In our survey, the most common recipients of cognitive enrichment were carnivores, supporting results of previous work looking at enrichment use [20]. These results show that carnivores, parrots, and primates most commonly receive cognitive enrichment however, mammals and birds are also the most housed animal groups [37]. This may cause these animal groups to be overrepresented in the survey due to their prevalence in zoos. The animal groups ‘fish’ and ‘amphibians’ were reported to receive cognitive enrichment, the least of which is unsurprising as these groups are underrepresented in the literature on cognitive enrichment [37]. Reptiles, recently shown to have a range of cognitive abilities thought to be exclusive to mammals and birds [38], had the highest percentage of respondents reporting that no cognitive enrichment is given even though they think it should be. It could be that the attitudes of zoo staff and enrichment use may evolve in response to scientific evidence, or vice versa. Regardless, there is a delay in the uptake of cognitive enrichment in reptiles that needs to be addressed.

Willingness alone to adopt new practice is not enough to ensure cognitive enrichment provision: time constraints, time to observe animals and limited finances were found to universally restrict the implementation of cognitive enrichment. Time constraints were also reported to impact the use of traditional enrichment [29]. This is expected as, to be able to deliver enrichment effectively, a 6-step process is suggested which involves research, planning, implementation, observation, evaluation, and readjustment [39]. When dealing with cognitive enrichment, additional needs for success also exist as information about the individual animal needs to be considered to ensure appropriate levels of challenge [11]. The time and design factors that were reported as restricting the use and success of cognitive enrichment come with an intrinsic financial cost. Staff time involved in cognitive enrichment development and review, particularly for species that previously have not received it, requires a financial investment from the zoo. These restrictions may be insurmountable regardless of the preference of staff to supply their animals with cognitive enrichment.

The fact that males and females only differed significantly in the rating of one factor, limited funds, suggests that sex does not have a large impact on responses. There were, however, regional variations in the perceived challenges of implementing successful cognitive enrichment. In North America, Oceania, and Europe time-related factors were more commonly reported as cognitive enrichment constraints; respondents from Asia rated considerations of workload for others highly and South and Central America sighted animal numbers as the most important constraint. Generally, respondents from Asia rated factors impacting the use of cognitive enrichment higher, most commonly contrasting with North America and Oceania. Some of this variation in rating could be attributed to differences in the way questions and scales are perceived across cultures [40,41]. Cultural differences may also play a role as culture and religion have been shown to influence people’s attitudes towards animals [42]. Other explanations for this difference could include geographical and language barriers between regions like Asia and North America that may make the transference of information and ideas is more difficult. This may contribute to more varied approaches in husbandry and enrichment practices than would be seen in regions more closely related. In light of these differences, strategies created to facilitate the use of cognitive enrichment should take into consideration the difficulties universally faced by the industry as well as factors perceived to be most important in the region which is being addressed.

This survey showed that cognitive enrichment relies heavily on the keepers. This high reliance on time-poor staff can be problematic for animals with more complex or unknown requirements. This is due to the level of knowledge needed to create appropriate challenge [21]. For keepers, it seems that obtaining this knowledge is done on the job or in their own time as 46.3% of respondents indicated that they have no formal education relevant to their current role. Keepers also must contend with differing views of management. This is illustrated by differences in how animal and management workers rated the factors ‘importance of enrichment to management/the workplace’ and ‘restriction/regulations on enrichment use’. Having knowledgeable staff who can specialize in enrichment and have open and informed discussions with all personnel involved in enrichment approval and management may result in more constant and appropriate cognitive enrichment. The addition of enrichment specialists would allow for a more in-depth understanding of the cognitive range of different species and the realistic needs of both the animals and the facility. This could allow for more species to be offered accessible enrichment with integrated cognitive challenge and further improve the welfare of animals in captivity.

The uncertainty around animal cognitive abilities and lack of agreement in what constitutes cognitive enrichment may contribute to the inconsistencies in classification seen in this survey. Cognition is not a single skill, it is a mental process involving the acquisition, storage, manipulation, and retrieval of information [11]. Cognitive capabilities are largely dictated by species [43] although the level of cognitive ability can be impacted by genetics [44], environmental complexity [45], personal experience [46] and social dynamics [47]. Published work discussing the creation of environmental enrichment highlights the need to take into consideration both the natural and individual history of the animal being enriched to achieve positive outcomes [39]. Within this framework there is no explicit mention of cognitive challenge, however, it has been proposed that its addition may be beneficial [48]. The broad definitions of cognitive enrichment coupled with the loose recommendations for enrichment creation leave it up to the individual to interpret what constitutes cognitive enrichment.

## 5. Conclusions

Cognitive enrichment is perceived as highly important to the welfare of animals amongst keepers, management, and other zoo staff, and the positive impact of its use is supported by scientific evidence. This survey showed that there is a need for clear definitions and guides around cognitive enrichment creation and use to aid in its adoption. Based on this survey, it is recommended that cognitive enrichment be incorporated more often and be delivered to more species than is currently reported. However, as the responsibility of implementing cognitive enrichment hinges on animal keepers who are already time-poor, changes to the current approach is required. To do this, cognitive enrichment must be made an integral component of animal care, not merely a positive addition if time allows. A financial investment from institutions in the form of staff time, focused training, detailed record keeping, and inter-institutional record sharing are required to ensure the continued improvement of captive animal welfare.

## Figures and Tables

**Table 1:** Factors affecting the use of cognitive enrichment that were presented to those who work with animals and those who do not, which have been combined for analysis.

Factors Combined	Do Not Work Directly with Animals	Work Directly with Animals
Limited training	Limited training opportunities for keepers to develop their enrichment skills	My limited knowledge about building enrichment
Limited knowledge	Limited keeper knowledge of enrichment principles	My personal knowledge of types of enrichment
Importance of enrichment to management/workplace	Limited management prioritization of enrichment	How important enrichment is to the company I work for
Time observe animal response	Restricted opportunity to observe animal response	My time to observe animal use of enrichment
Limited funds	Limited funds available for enrichment	My limited access to funds to build or buy enrichment
Time constraints	Time constraints	The extent to which I have more pressing duties to complete
Past enrichment failures	Past enrichment failures	My personal experience with past enrichment failures
Restrictions/regulation on enrichment	Management restrictions on certain types of enrichment	The zoo regulations of enrichment practices I must follow
Concerns around unnatural enrichment	Restrictions on “unnatural” enrichment	Concerns that the public will respond negatively to unnatural enrichment
Number of animals	Keeper to animal ratio	The number of different animals I need to provide enrichment for
Workload for others	Workload for colleagues or other keepers	The effect the enrichment will have on other keepers’ workload

**Table 2:** Demographic information for respondents included in analysis. The number of respondents in each category are presented with the proportion of the total shown as a percentage in parenthesis.

	Asia	Europe	North America	Oceania	South/Central America	Focal Zoo	Total
*n*	53 (29.9)	23 (13)	24 (13.6)	19 (10.7)	6 (3.4)	52 (29.4)	177 (100)
**Sex**							
Female	26 (14.7)	16 (9)	20 (11.3)	13 (7.3)	4 (2.3)	36 (20.3)	115 (65)
Male	24 (13.6)	6 (3.4)	4 (2.3)	6 (3.4)	2 (1.1)	14 (7.9)	56 (31.6)
Not provided	2 (1.1)	1 (0.6)	0 (0)	0 (0)	0 (0)	2 (1.1)	5 (2.8)
Other	1 (0.6)	0 (0)	0 (0)	0 (0)	0 (0)	0 (0)	1 (0.6)
**Age**							
18–24	11 (6.2)	3 (1.7)	3 (1.7)	2 (1.1)	0 (0)	3 (1.7)	22 (12.4)
25–34	23 (13)	10 (5.6)	11 (6.2)	8 (4.5)	3 (1.7)	15 (8.5)	70 (39.5)
35–44	16 (9)	7 (4)	7 (4)	3 (1.7)	2 (1.1)	16 (9)	51 (28.8)
45–54	3 (1.7)	3 (1.7)	3 (1.7)	5 (2.8)	1 (0.6)	9 (5.1)	24 (13.6)
55–64	0 (0)	0 (0)	0 (0)	1 (0.6)	0 (0)	6 (3.4)	7 (4)
65–74	0 (0)	0 (0)	0 (0)	0 (0)	0 (0)	2 (1.1)	2 (1.1)
Not provided	0 (0)	0 (0)	0 (0)	0 (0)	0 (0)	1 (0.6)	1 (0.6)
**Current role**							
Animal worker	32 (18.1)	16 (9)	15 (8.5)	13 (7.3)	4 (2.3)	28 (15.8)	108 (61)
Management	4 (2.3)	5 (2.8)	7 (4)	6 (3.4)	2 (1.1)	10 (5.6)	34 (19.2)
Non Animal worker	17 (9.6)	2 (1.1)	2 (1.1)	0 (0)	0 (0)	14 (7.9)	35 (19.8)
**Experience in current role**							
1–5 years	32 (18.1)	13 (7.3)	13 (7.3)	7 (4)	6 (3.4)	26 (14.7)	97 (54.8)
6–10 years	9 (5.1)	2 (1.1)	6 (3.4)	6 (3.4)	0 (0)	9 (5.1)	32 (18.1)
11–15 years	4 (2.3)	4 (2.3)	1 (0.6)	1 (0.6)	0 (0)	3 (1.7)	13 (7.3)
16–20 years	3 (1.7)	2 (1.1)	2 (1.1)	1 (0.6)	0 (0)	5 (2.8)	13 (7.3)
20+ years	3 (1.7)	2 (1.1)	2 (1.1)	4 (2.3)	0 (0)	9 (5.1)	20 (11.3)
Not provided	2 (1.1)	0 (0)	0 (0)	0 (0)	0 (0)	0 (0)	2 (1.1)
**Highest level of education**							
Less than high school	0 (0)	1 (0.6)	0 (0)	0 (0)	0 (0)	0 (0)	1 (0.6)
High school graduate	3 (1.7)	2 (1.1)	2 (1.1)	1 (0.6)	0 (0)	1 (0.6)	9 (5.1)
Technical collage/Trade qualification	5 (2.8)	7 (4)	0 (0)	3 (1.7)	0 (0)	15 (8.5)	30 (16.9)
Undergraduate degree	31 (17.5)	5 (2.8)	19 (10.7)	10 (5.6)	2 (1.1)	27 (15.3)	94 (53.1)
Masters degree	12 (6.8)	5 (2.8)	2 (1.1)	3 (1.7)	3 (1.7)	9 (5.1)	34 (19.2)
Doctorate	2 (1.1)	3 (1.7)	1 (0.6)	2 (1.1)	1 (0.6)	0 (0)	9 (5.1)
**Relevant education**							
No relevant education	19 (10.7)	14 (7.9)	13 (7.3)	11 (6.2)	4 (2.3)	21 (11.9)	82 (46.3)
Technical collage/Trade qualification	7 (4)	9 (5.1)	0 (0)	3 (1.7)	0 (0)	17 (9.6)	36 (20.3)
Undergraduate degree	19 (10.7)	0 (0)	11 (6.2)	3 (1.7)	1 (0.6)	13 (7.3)	47 (26.6)
Masters degree	7 (4)	0 (0)	0 (0)	2 (1.1)	1 (0.6)	1 (0.6)	11 (6.2)
Doctorate	1 (0.6)	0 (0)	0 (0)	0 (0)	0 (0)	0 (0)	1 (0.6)

**Table 3:** Median response to “How important do you think the following types of enrichment are to the welfare of captive animals?”. Responses were categorized by region, using a Likert scale of 1–7: 1 = Not important and 7 = very important. Standard error is presented in parenthesis.

Enrichment Type	Asia	Europe	North America	Oceania	South/Central America	Focal Zoo	Global *
Cognitive Enrichment	7 (0.13)	7 (0.12)	7 (0.04)	7 (0.1)	7 (0.33)	7 (0.08)	7 (0.05)
Feeding Enrichment	7 (0.18)	7 (0.06)	7 (0.19)	7 (0.12)	7 (0)	7 (0.12)	7 (0.07)
Structural Enrichment	7 (0.11)	7 (0.16)	7 (0.14)	7 (0.19)	7 (0)	7 (0.17)	7 (0.07)
Social Enrichment	7 (0.18)	7 (0.11)	7 (0.23)	7 (0.22)	7 (0)	7 (0.1)	7 (0.08)
Tactile Enrichment	6 (0.17)	6 (0.25)	6 (0.25)	7 (0.26)	6 (0.54)	7 (0.17)	7 (0.09)
Olfactory Enrichment	6 (0.19)	7 (0.2)	6.5 (0.22)	7 (0.3)	7 (0.49)	7 (0.14)	7 (0.09)
Visual Enrichment	6 (0.17)	6 (0.28)	6 (0.28)	7 (0.24)	5.5 (0.76)	7 (0.18)	6 (0.1)
Auditory Enrichment	6 (0.21)	4 (0.4)	5.5 (0.37)	6 (0.38)	5.5 (0.67)	6 (0.2)	6 (0.12)
Human-Animal Interaction	5 (0.24)	4 (0.29)	7 (0.34)	5 (0.39)	6.5 (0.71)	5 (0.21)	5 (0.12)

* Inclusive of all regions. Asia, Europe, North America, Oceania, South/Central America, and Focal zoo.

**Table 4:** The number of respondents, by region and globally, that reported which animal groups receive cognitive enrichment (CE) within their facility; with the final column showing the percentage of those animal groups which respondents feel should be receiving cognitive enrichment but currently do not. Animal groups organized according to global total in descending order. Percentage is presented in parenthesis.

Animal Group	Global Total *	ASIA	Europe	North America	Oceania	South/Central America	Focal Zoo	Not Receiving CE but Should *
Carnivores	135 (76.3)	37 (69.8)	18 (78.3)	20 (83.3)	11 (57.9)	5 (83.3)	44 (84.6)	60 (33.9)
Primates (exclusive of great apes)	126 (71.2)	33 (62.3)	20 (87)	21 (87.5)	8 (42.1)	4 (66.7)	40 (76.9)	54 (30.5)
Parrots	105 (59.3)	23 (43.4)	11 (47.8)	18 (75)	15 (78.9)	5 (83.3)	33 (63.5)	68 (38.4)
Great apes	98 (55.4)	26 (49.1)	13 (56.5)	11 (45.8)	5 (26.3)	2 (33.3)	41 (78.8)	55 (31.1)
Small Carnivores	94 (53.1)	19 (35.8)	11 (47.8)	20 (83.3)	6 (31.6)	4 (66.7)	34 (65.4)	57 (32.2)
Other birds	87 (49.2)	19 (35.8)	9 (39.1)	14 (58.3)	11 (57.9)	4 (66.7)	30 (57.7)	71 (40.1)
Elephants	79 (44.6)	27 (50.9)	9 (39.1)	5 (20.8)	3 (15.8)	3 (50)	32 (61.5)	52 (29.4)
Ungulates	75 (42.4)	15 (28.3)	9 (39.1)	16 (66.7)	6 (31.6)	4 (66.7)	25 (48.1)	62 (35.0)
Reptiles	72 (40.7)	11 (20.8)	4 (17.4)	14 (58.3)	11 (57.9)	3 (50)	29 (55.8)	79 (44.6)
Marsupials and monotremes	71 (40.1)	11 (20.8)	4 (17.4)	10 (41.7)	13 (68.4)	1 (16.7)	32 (61.5)	55 (31.1)
Marine Mammals	62 (35)	12 (22.6)	8 (34.8)	5 (20.8)	6 (31.6)	2 (33.3)	29 (55.8)	56 (31.6)
Corvids	47 (26.6)	6 (11.3)	5 (21.7)	14 (58.3)	2 (10.5)	2 (33.3)	18 (34.6)	55 (31.1)
Fish	30 (16.9)	4 (7.5)	4 (17.4)	4 (16.7)	5 (26.3)	1 (16.7)	12 (23.1)	67 (37.9)
Amphibians	30 (16.9)	5 (9.4)	3 (13)	6 (25)	3 (15.8)	0 (0)	13 (25)	63 (35.6)
I do not know	18 (10.2)	5 (9.4)	1 (4.3)	2 (8.3)	0 (0)	0 (0)	10 (19.2)	(0.0)

* Inclusive of all regions. Asia, Europe, North America, Oceania, South/Central America, and Focal zoo.

**Table 5:** Average Likert rating for factors affecting the use of cognitive enrichment within region and job role with standard error in parenthesis. 1 = No effect to 7 = Significant effect. Statistically significant differences in means within factor (e.g., number of animals) are indicated with alphanumerical superscripts. Regions, alphabetical (A, B, C) and or work role, numerical (1, 2).

Factor	Asia	Europe	North America	Oceania	South/Central America	Focal Zoo	Work Directly with Animals	Management	Do Not Work Directly with Animals	Gobal Average *
Time constraints	5.3 (0.20)	5.9 (0.33)	5.5 (0.33)	4.9 (0.38)	6.3 (0.42)	5.4 (0.23)	5.4 (0.15)	5.6 (0.26)	5.2 (0.25)	5.4 (0.12)
Time observe animal response	5.4 (0.19)	4.7 (0.45)	4.8 (0.31)	4.9 (0.36)	6.5 (0.50)	5.1 (0.22)	5.2 (0.16)	5.1 (0.29)	5.1 (0.26)	5.1 (0.12)
Limited funds	5.2 (0.25)	4.7 (0.46)	4.4 (0.45)	4.7 (0.45)	3.8 (0.79)	5.4 (0.23)	5 (0.19) ^12^	4.2 (0.33) ^1^	5.7 (0.26) ^2^	5.0 (0.14)
Number of animals	5.5 (0.23) ^BC^	5.1 (0.44) ^ABC^	4.8 (0.37) ^ABC^	4.0 (0.50) ^AB^	6.7 (0.21) ^C^	4.2 (0.26) ^A^	4.9 (0.2)	4.6 (0.31)	4.7 (0.3)	4.8 (0.15)
Workload for others	5.5 (0.20) ^A^	4.6 (0.40) ^AB^	3.6 (0.41) ^B^	3.4 (0.45) ^B^	4.7 (1.12) ^AB^	4.5 (0.27) ^AB^	4.4 (0.19)	4.5 (0.36)	5.1 (0.29)	4.6 (0.15)
Importance of enrichment to management/ workplace	5.3 (0.24) ^A^	3.2 (0.47) ^B^	3.1 (0.44) ^B^	3.8 (0.47) ^AB^	4.8 (0.95) ^AB^	3.4 (0.27) ^B^	4.3 (0.21) ^1^	2.9 (0.32) ^2^	4.5 (0.34) ^12^	4.0 (0.16)
Restrictions regulation on enrichment	4.7 (0.25) ^A^	2.4 (0.33) ^B^	3.4 (0.45) ^AB^	2.9 (0.38) ^B^	3.8 (0.95) ^AB^	4.8 (0.23) ^A^	4.2 (0.19) ^1^	3.1 (0.31) ^2^	4.5 (0.31) ^12^	4.0 (0.15)
Limited knowledge	4.9 (0.25) ^A^	3.7 (0.28) ^AB^	2.8 (0.32) ^B^	3.0 (0.29) ^B^	4.8 (0.79) ^AB^	3.4 (0.25) ^B^	3.8 (0.18)	3.8 (0.3)	3.9 (0.32)	3.8 (0.14)
Limited training	4.6 (0.29) ^A^	3.3 (0.38) ^AB^	2.7 (0.35) ^B^	2.4 (0.29) ^B^	4.2 (0.65) ^AB^	3.4 (0.28) ^B^	3.5 (0.19)	3.3 (0.36)	3.9 (0.35)	3.6 (0.15)
Concerns around unnatural enrichment	4.4 (0.28) ^A^	2.3 (0.35) ^B^	2.7 (0.38) ^B^	2.7 (0.39) ^B^	3.7 (1.15) ^AB^	3.8 (0.27) ^AB^	3.2 (0.21)	3.7 (0.32)	4.3 (0.27)	3.5 (0.16)
Past enrichment failures	4.2 (0.28) ^A^	3.0 (0.37) ^AB^	2.5 (0.35) ^B^	2.5 (0.42) ^AB^	4.2 (1.01) ^AB^	3.2 (0.23) ^AB^	3.1 (0.2) ^1^	3.1 (0.27) ^12^	4.3 (0.29) ^2^	3.3 (0.15)

* Inclusive of all regions. Asia, Europe, North America, Oceania, South/Central America, and Focal zoo.

**Table 6:** Average Likert rating for factors affecting the success of cognitive enrichment within regions with standard error in parenthesis. 1 = No effect to 7 = Significant effect. Statistically significant differences in means between regions for each factor (e.g., Animal intelligence) are indicated with alphanumerical superscripts (A, B).

Factor	Asia	Europe	North America	Oceania	South/Central America	Focal Zoo	Global Average *
Keeper interest	6.1 (0.14)	5.7 (0.36)	5.5 (0.36)	5.3 (0.39)	6.7 (0.33)	5.8 (0.21)	5.8 (0.11)
Time constraints	5.2 (0.23)	6.1 (0.22)	6.1 (0.25)	5.3 (0.37)	6.2 (0.48)	5.7 (0.24)	5.6 (0.12)
Enrichment design	4.7 (0.24) ^A^	5.5 (0.33) ^AB^	5.6 (0.27) ^AB^	5.8 (0.34) ^AB^	6.7 (0.33) ^AB^	6.3 (0.15) ^B^	5.6 (0.12)
Level of difficulty for keepers	5.3 (0.17) ^A^	5.2 (0.35) ^AB^	4.9 (0.34) ^AB^	4.2 (0.33) ^B^	4.8 (0.70) ^AB^	5.6 (0.22) ^A^	5.2 (0.12)
Animal personality	5.3 (0.23)	4.8 (0.39)	5.0 (0.39)	5.4 (0.28)	5.0 (0.63)	4.8 (0.26)	5.0 (0.13)
Animal intelligence	5.3 (0.25) ^A^	4.5 (0.47) ^AB^	4.4 (0.39) ^AB^	5.3 (0.32) ^A^	4.2 (0.87) ^AB^	3.8 (0.28) ^B^	4.6 (0.15)
Visitor interest	4.3 (0.23) ^A^	2.4 (0.34) ^B^	2.5 (0.31) ^B^	2.8 (0.36) ^B^	1.5 (0.50) ^B^	2.3 (0.21) ^B^	3.0 (0.14)
Animal sex	3.5 (0.22) ^A^	1.9 (0.34) ^B^	1.5 (0.20) ^B^	2.0 (0.29) ^B^	1.7 (0.49) ^AB^	1.8 (0.17) ^B^	2.3 (0.12)

* Inclusive of all regions. Asia, Europe, North America, Oceania, South/Central America, and Focal zoo.

## Data Availability

The data presented in this study are available on request from the corresponding author. The data are not publicly available due to privacy of respondents.
